# FDM 3D Printed Composites for Bone Tissue Engineering Based on Plasticized Poly(3-hydroxybutyrate)/poly(d,l-lactide) Blends

**DOI:** 10.3390/polym12122806

**Published:** 2020-11-27

**Authors:** Veronika Melčová, Kateřina Svoradová, Přemysl Menčík, Soňa Kontárová, Michala Rampichová, Věra Hedvičáková, Věra Sovková, Radek Přikryl, Lucy Vojtová

**Affiliations:** 1 Institute of Material Chemistry, Faculty of Chemistry, Brno University of Technology, Purkyňova 464/118, 612 00 Brno, Czech Republic; xcsvoradova@vutbr.cz (K.S.); mencik@fch.vut.cz (P.M.); kontarova@fch.vut.cz (S.K.); prikryl@fch.vut.cz (R.P.); 2 Institute of Experimental Medicine, Czech Academy of Sciences, Vídeňská 1083, 142 20 Prague 4, Czech Republic; Michala.rampichova@iem.cas.cz (M.R.); vera.lukasova@iem.cas.cz (V.H.); vera.sovkova@iem.cas.cz (V.S.); 3 CEITEC—Central European Institute of Technology, Brno University of Technology, Advanced Biomaterials, Purkyňova 656/123, 612 00 Brno, Czech Republic

**Keywords:** additive manufacturing, fused deposition modeling, poly(3-hydroxybutyrate), polylactide, tricalcium phosphate, regenerative medicine, tissue engineering, bone scaffolds

## Abstract

Tissue engineering is a current trend in the regenerative medicine putting pressure on scientists to develop highly functional materials and methods for scaffolds’ preparation. In this paper, the calibrated filaments for Fused Deposition Modeling (FDM) based on plasticized poly(3-hydroxybutyrate)/poly(d,l-lactide) 70/30 blend modified with tricalcium phosphate bioceramics were prepared. Two different plasticizers, Citroflex (*n*-Butyryl tri-*n*-hexyl citrate) and Syncroflex (oligomeric adipate ester), both used in the amount of 12 wt%, were compared. The printing parameters for these materials were optimized and the printability was evaluated by recently published warping test. The samples were studied with respect to their thermal and mechanical properties, followed by biological in vitro tests including proliferation, viability, and osteogenic differentiation of human mesenchymal stem cells. According to the results from differential scanning calorimetry and tensile measurements, the Citroflex-based plasticizer showed very good softening effect at the expense of worse printability and unsatisfactory performance during biological testing. On the other hand, the samples with Syncroflex demonstrated lower warping tendency compared to commercial polylactide filament with the warping coefficient one third lower. Moreover, the Syncroflex-based samples exhibited the non-cytotoxicity and promising biocompatibility.

## 1. Introduction

Skeletal damage is among the most common health issues, mostly originating from osteoporosis and traumatic fractures [1,2]. A huge effort of scientists and commercial sector is applied to an investigation of new efficient and patient friendly treatment methods, such as tissue engineering.

Tissue engineering (TE) is one of the modern concepts in regenerative medicine. This multi-disciplinary approach connects the knowledge from the fields of medicine and biology with material sciences. In TE, the scaffold as a supportive material, which may be seeded with cells and/or other supplementary factors, is prepared in vitro and used as an implant for affected tissue regeneration. In orthopedics, TE finds application when the extent of the bone injury excludes the self-healing by natural processes of ossification. At the same time, it should offer superior performance to other methods of regenerative medicine exploited in such cases, as the bone autographs or allografts [3,4,5,6].

A suitable 3D structure needs to be ensured so that the entire interior of a scaffold is accessible for cells’ migration regardless of whether the material is cultivated with cells in vitro or the cells originate from the patient’s body [7]. Polymeric scaffolds for tissue engineering with such 3D structures can be technologically prepared in several ways, such as by casting a solution or a melt into molds, by lyophilization, foaming, eluting porogenic particles, or by various spinning techniques [7,8,9,10]. Currently, a relatively new fabrication method of 3D printing (known as additive manufacturing) is also being successfully used. 3D printed product is created layer by layer according to an accurate digital 3D model. Hence the biggest advantage of this method is the ability to produce time and cost effectively complex shapes, including porous internal structures [11].

There are several 3D printing techniques which use different types of input materials and are reviewed elsewhere [12]. For the preparation of scaffolds for bone tissue replacement, the use of Selective Laser Sintering (SLS) method, where a powder is fusioned by laser beam, was published [13,14,15]. 3D printing technique used in this article is based on the Fused Deposition Modeling (FDM) in which a plastic filament of exact diameter is led to a heated nozzle, where it is melted and extruded onto a substrate, where it forms the first layer of formed object. This method becomes increasingly popular mainly due to its low cost, high speed, and simplicity [16,17,18]. Common materials for FDM printers are mainly polylactide (PLA) and acrylonitrile butadiene styrene (ABS), but other conventional polymers, composites and biopolymers are also getting more attention at present [19,20,21,22].

Disk shaped scaffolds were successfully prepared by FDM from semi-crystalline PLA and seeded with human fetal osteoblasts. According to the authors, FDM has not affected the biocompatibility of PLA in any way, although the cell growth was more pronounced in the control sample (polystyrene) than in PLA. This may be related to the resulting surface of the scaffold, which was not optimized in this work [23]. The first studies focusing on FDM 3D printing of biodegradable polyhydroxyalkanoates (PHA) have also appeared. PHA are a group of microbial polyesters which are biocompatible and biodegradable and therefore promising candidates for a scaffold material [24,25]. Recently, FDM printed scaffolds from poly(3-hydroxybutyrate-*co*-3-hydroxyhexanoate) (PHBH) were evaluated regarding their thermal, rheological, and mechanical properties. In addition, their cytocompatibility with mouse embryonic fibroblast cells and the biodegradation in synthetic gastric juice were tested, showing overall better results than PLA scaffolds [26]. Moreover, poly(3-hydroxybutyrate-3-hydroxyvalerate) (PHBV) another widely studied representative of PHA group, was blended with PLA and used for 3D printing of dumbbell specimens for mechanical tests. The material was shown to be non-toxic toward human embryonic kidney cells and normal human lung fibroblasts [27].

With a great success, composite materials combining the advantage of easy polymer processability and ceramic filler bioactivity are often investigated for TE scaffolds. Hydroxyapatite (HA), natural inorganic component of bone, and tricalcium phosphate (TCP) are two most abundant examples. These fillers were reported to be applicable for FDM 3D printing in PLA [28] or polycaprolactone (PCL) matrix [29,30,31]. Although HA has the closest chemical structure to that of a bone mineral, it is the least soluble of calcium phosphate ceramics used. Moreover, stochiometric HA is osteoconductive, but not osteoinductive unlike TCP, which possesses both these properties. At the same time, TCP is more soluble and therefore its biosorption is faster [32]. Konopnicky et al. studied 3D printed constructs made from TCP filled PCL (50 wt.% of the filler) seeded with porcine bone marrow progenitor cells to treat man-made mandible defects in minipigs. Eight weeks after the surgery, the seeded scaffolds showed better bone penetration depth than the controls [30].

With this article we wish to contribute to the common goal of improvement of current treatment methods by exploiting functional, biodegradable polymers and additives and state-of-the-art preparation method. The goal is to develop a material with suitable thermal and mechanical properties to be processed by FDM 3D printing method. At the same time, this material has to meet all the conditions for being used in vivo in tissue engineering of bones namely to be osteoconductive, osteoinductive, osteogenic, resorbable or degradable [3,6]. To achieve this, we used the following materials: (i) poly(3-hydroxybutyrate) (PHB), the most published and commercially successful PHA polymer, which has all the above-mentioned features of PHA family; (ii) amorphous PLA, which contributes to the processability and mechanical properties of the resulting material; (iii) plasticizers varying in molecular weight and chemical structure to further improve overall properties and printability; and (iv) TCP to promote a good response of the materials in vivo. Our research group drawn from its unique experience with FDM 3D printing of PHB/PLA blends [33,34].

## 2. Materials and Methods

### 2.1. Preparation of Composites

Four composite samples based on plasticized blend of poly(3-hydroxybutyrate) Y1000P from TianAn Biologic Materials (Ningbo City, China, abbreviated PHB) and amorphous poly(d,l-lactide) Ingeo 4060D from NatureWorks (Minnetonka, MN, USA, abbreviated PLA) were used for all studies. Citroflex® B-6 (*n*-Butyryl tri-*n*-hexyl citrate, abbreviated as CT) from Vertellus Holdings LLC Company (Indianapolis, IN, USA) and Syncroflex TM 3114 (oligomeric adipate ester, referred as abbr. SN) from Croda (Rawcliff, UK) were used as plasticizers. Tricalcium phosphate blend (αTCP = 7%, βTCP = 93%, abbr. TCP) (CN Lab Nutrition, Shaanxi, China) was added to the matrix as a bioactive filler. The mean particle size of TCP is 10.8 µm as measured by laser diffraction using HELOS analyzer by Sympatec GmbH (Remlingen, Germany).

The composition of prepared biocomposites is given by Table 1. All samples were compounded using the corotating meshing twin screw extruder, from Labtech Engineering Company (Samutprakarn, Thailand, D = 16 mm, and L/D = 40). The temperature setting of individual zones of the extruder was 160–175–175–175–175–175–175–170–165–160 °C from a hopper to a nozzle and the rotational speed of screw was set to 130 rev.∙min^−1^. The total weight of each sample for extrusion was 500 g.

### 2.2. Thermal Characterization 

#### 2.2.1. Differential Scanning Calorimetry

Differential scanning calorimetry (DSC) measurements were performed on DSC 2500 (TA Instruments, New Castle, DE, USA). The samples were subjected to two heating runs from 10 to 195 °C with the heating rate of 10 °C∙min^−1^. All measurements were carried out under the nitrogen atmosphere. The polymer sample masses were approximately 10 mg. Aluminum pans with samples were hermetically sealed before the measurement. The crystallinity was calculated from DSC measurement according the equation:

(1) $$X_{c}=\frac{\Delta H_{m}}{\Delta H_{m}^{0}}\cdot 100\%,$$

where Δ*H_m_*, and Δ*H*^0^*_m_* (J/g) are the enthalpy of fusion from the second heating cycle and the enthalpy of fusion of 100% crystalline polymer (146 J/g for PHB), respectively. 

#### 2.2.2. Thermogravimetric Analysis

The curves from thermogravimetric analysis (TGA) were obtained using Q500 TG analyzer (TA Instruments, New Castle, DE, USA). After the equilibration at 40 °C, the samples were heated to 550 °C at the heating rate of 10 °C∙min^−1^ under nitrogen atmosphere. Subsequently, the temperature ramp continued to 600 °C at the same heating rate under air atmosphere.

### 2.3. Preparation of 3D Printing Filaments

Granules prepared in the first step were processed using the HAAKE™ Rheomex OS single screw extruder (Haake Technik GmbH, Vreden, Germany) into the form of 3D printing filaments. The chamber temperature profile from a hopper to a nozzle was set to 185–175–170–150 °C and the rotational speed of screw was kept 25 rev.∙min^−1^. The filament was firstly pulled into the water tank tempered to 60 °C. Secondly, the filament was directed to the draw-off device with the calibration unit in order to ensure a constant diameter of the filament of 1.75 mm.

### 2.4. 3D Printing and Printability Tests

Original Prusa i3 MK3 3D printer (Prusa Research, Prague, Czech Republic) was used for all studies. Maximum printing space of this printer is 250 × 210 × 200 mm (*x* × *y* × *z*), the resolution of *x* and *y* axes is 10 μm and the resolution of *z* axis is 5 μm. The basic printing parameters of used printer are given in Table 2.

Prepared biocomposites underwent the tests especially designated by our research group for the purpose of evaluating the printability. Firstly, the temperature of the nozzle was optimized by the Temperature Tower Test (TTT). In this test, two towers composed of several identical floors containing different geometrical elements are used. Each floor is printed with different temperature, which allows us to visually evaluate the quality of the print and the optimal printing temperature. Further details can be found in our previous publication [33].

Afterwards, the filling study aiming at proper setting of the parameter flow—volume passed through the extruder, was conducted to ensure optimal density and compactness of 3D printed specimens and therefore meaningful mechanical properties. A crucial factor to which an attention must be paid is the filament diameter. If the diameter is higher than desired 1.75 mm, the printer feeds excessive material which leads to the deterioration of mechanical properties and specimen surface or even to nozzle clogging. Opposite to this, thinner filament will cause the specimen to be underfilled with voids between individual perimeters. Such a sample will not be sintered properly which will manifest during the mechanical tests. Therefore, three samples (bars with dimensions of 30 × 10 × 4 mm) with different setting of flow value were printed. Typically, 95, 100 and 105% of flow was used for all samples and a broader range was studied when necessary. Samples were frozen (−18 °C) and observed using SZ51 optical microscope from Olympus (Tokyo, Japan).

Last but not least, warping—a negative phenomenon occurring during 3D printing was quantified using the printing conditions optimized by previous tests (two temperatures and one value of flow). In the warping test, the specimen consists of square platform on which a V-shaped beam adhering to bed only by its edge is connected. During printing, warping causes the front part beam to lift and detach from the bed. The maximum printed height before this happens is recorded and used for the calculation of the warping coefficient:

(2) $$warping\;coefficient=\;\frac{theoretical\;height\;of\;warping\;test\;specimen\;\left(10\;\mathrm{mm}\right)}{height\;of\;warping\;test\;specimen\;achieved\;during\;printing}$$

The test is thoroughly described in our previous publication [33]. The results are shown as mean ± standard deviation (SD) from five testing specimens.

### 2.5. Mechanical Testing

#### 2.5.1. Tensile Test

Standardized double-paddle testing specimens (dogbones 5A with cross section 4 × 2 mm, according to the CSN EN ISO 527) were 3D printed from prepared filaments. The G-code for printing the dogbones was made by regular alternating of two layers (i) the first layer is formed by simple import of fabricated model to original PrusaSlicer software (Prusa Research, Prague, Czech Republic) with adjusting the perimeters to form the entire neck of the dogbone and (ii) the second layer is formed manually by inserting infill parallel to the longest dimension of the dogbone. The development of this G-code is described in our previous work [34]. The Zwick-Roel Z010 (ZwickRoell GmbH & Co., Ulm, Germany) device with load indicator of maximum tensile force 1 kN and pneumatic grips with 2.5 kN maximum gripping force were used for the measurements. The deformation rates for the determination of Young’s elastic modulus and for the rest of the test were set to 5 mm·min^−1^ and 50 mm∙min^−1^, respectively. However, the Young’s modulus was determined without the extensometer. All measurements were carried out 10 days after the sample preparation to ensure that the material aging process after the preparation is finished. The elongation at break, the Young´s modulus, and the tensile strength were determined as the average of at least six measurements, and the results are shown as mean ± SD.

#### 2.5.2. Flexure Test

The three-point bending test of studied materials was performed according to the CSN EN ISO 178 standard method A. Rectangular testing specimens with dimensions of 80 × 10 × 4 mm were 3D printed from prepared filaments. The G-code for 3D printing was created in original PrusaSlicer software (Prusa Research, Prague, Czech Republic) so that the entire volume of the specimen was composed from perimeters. The Zwick-Roel Z010 (ZwickRoell GmbH & Co., Ulm, Germany) device with load indicator of maximum force 1 kN was used for the measurements. The radius of loading nose and supports was 5 mm, the support span was 64 mm. The flexural modulus was determined from the deformation range of 0.05–0.25%. The test speed was set to 2 mm∙min^−1^. A limit deformation of 5% was also set. The results in the graph are mean average from six samples with error bars from SD.

### 2.6. In Vitro Tests on Scaffolds

The granules of prepared composites were compression molded in a laboratory hot press to obtain plates with the thickness of approximately 1 mm. Round specimens with the diameter of 6 mm were cut off from these plates for 96 well plates (MTS assay, DNA quantification, ALP activity measurement) and with the diameter of 10 mm for 48 well plates (confocal microscopy and PCR analysis). Prepared scaffolds were sterilized (Military University Hospital Prague) with ethylene dioxide and seeded with human mesenchymal stem cells (hMSC, Sciencell, Carlsbad, CA, USA). To enhance an osteogenic differentiation of cells, used culture medium contained Eagle’s minimal essential medium (α-MEM) with 10% of fetal bovine serum (FBS), 1% of penicillin/streptomycin, 100 nM of dexamethasone, 10 mM of β-glycerol phosphate and 50 μg∙mL^−1^ of ascorbic acid-2-phosphate. Cells were seeded in the density of 100 × 10^3^ hMCS in 125 μL of culture medium on 48 well plate and 15 × 10^3^ hMSC in 41 μL of culture medium on 96 well plates. After two hours of adhesion, the medium was filled to the volume of 750 μL in the case of 48 well plate and 250 μL in the case of 96 well plate. hMSCs were seeded also on the bottom of the 96 well plates for some assays (tissue culture polystyrene abbreviated PS). Half of the medium was changed every week. MTS test, DNA quantification, osteogenic differentiation quantification and visualization of cells were conducted after 1, 7, 14, 21, and 28 days of the experiment.

#### 2.6.1. Metabolic Activity Assay

The scaffolds were transferred to the clean plates and 100 μL of growth medium (without osteogenic supplements) and 20 μL of MTS substrate((3-(4,5-dimethylthiazol-2-yl)-5-(3-carboxy-methoxyphenyl)-2-(4-sulfo-phenyl)-2H-tetrazolium) (Sigma Aldrich, St. Louis, MO, USA) was added. After two hours of incubation at 37 °C, the amount of formazan formed by metabolizing the MTS substrate was determined spectrophotometrically from 100 μL transferred to a new well plate. The absorbance of the solutions was measured at 490 nm with 690 nm as a reference wavelength using the microplate reader Infinite® M200 PRO from Tecan (Männedorf, Switzerland). The absorbance of the MTS solution incubated with scaffolds without seeded cells was subtracted from the values.

#### 2.6.2. DNA Quantification

The proliferation of the cells on scaffolds was determined using DNA quantification. After MTS measurement, the scaffolds were transferred into 200 μL of lysis buffer composed of 10 mM tris(hydroxymethyl) aminomethane (Tris), 1mM ethylenediamine-tetraacetic acid (EDTA), 0.2% Triton-X-100 and distilled water. Subsequently, the samples were subjected to three freeze/thaw cycles, between which the samples were roughly vortexed. 200 μL of Quant-iT™ dsDNA Assay Kit reagent and 10 μL of cell lysate were added to 96 well plate. The fluorescence of the reagent after the reaction with DNA from the lysate was measured using the multiplate fluorescence reader Infinite® M200 PRO from Tecan (Männedorf, Switzerland) at *λ*_ex_ = 485 nm and *λ*_em_ = 528 nm. The DNA content was determined according to the calibration curve using the standards in the kit.

#### 2.6.3. Cell Visualization

The fluorescent staining and confocal microscopy were used for the cell visualization. The samples were fixed with frozen methyl alcohol (−20 °C) for 10 min and rinsed with phosphate buffered saline (PBS, pH 7,4). Afterwards, the samples were incubated with DiOC6(3) (3,3’-diethyl-hexacarbocyanine iodide, Invitrogen, Molecular Probes, 1 μg∙mL^−1^ in PBS) at room temperature for 45 min. For the nuclei visualization, the incubation of samples with propidium iodide (Sigma Aldrich, St. Louis, MO, USA, 5 μg∙mL^−1^ in PBS) followed. Subsequently, the samples were rinsed with PBS three times and observed using the LSM 5 DUO confocal microscope Zeiss (Oberkochen, Germany). Used wavelengths were *λ*_ex_ = 488 nm and *λ*_em_ = 520 nm in the case of DiOC6(3) and *λ*_ex_ = 560 nm and *λ*_em_ = 580 nm in the case of propidium iodide.

#### 2.6.4. Alkaline Phosphatase Activity

Alkaline phosphatase (ALP), being an early marker of osteogenic differentiation, was determined on days 1, 7, 14, and 21 of the experiment. The scaffolds were rinsed in PBS and incubated at room temperature for 30 min with 100 μL of ALP substrate (p-nitrophenyl phosphate liquid substrate system; Sigma Aldrich, St. Louis, MO, USA). After 30 min, the reaction was stopped by adding 50 μL of 2M NaOH and the amount of produced p-nitrophenol was determined by measuring the absorbance at 405 nm with the microplate reader Infinite® M200 PRO from Tecan (Männedorf, Switzerland). The absorbance of ALP substrate incubated with scaffolds without seeded cells was subtracted from the values.

#### 2.6.5. Osteogenic Differentiation Analysis

The samples were transferred to a 1.5 mL test tube with lysis buffer (RLT buffer) and crushed with a tweezer. RNA of the samples was isolated using the Qiagen RNeasy Mini Kit (Hilden, Germany) according to the manufacturer’s protocol. The RNA concentration was determined spectrophotometrically as the absorbance at 260/280 nm with the microplate reader Infinite® M200 PRO from Tecan (Männedorf, Switzerland). Subsequently, cDNA was synthetized using the RevertAid H Minus First Strand cDNA Synthesis Kit from Thermo Scientific (Waltham, MA, USA). The real-time polymer chain reaction (RT-PCR) was performed to multiply the amount of cDNA. The gene sequences for osteogenic markers RunX2 (86 bp, RUNX2, Hs01047973_m1, Thermo Scientific), type I collagen (66 bp, COL1A1 Hs00164004_m1,Thermo Scientific), osteocalcin (138 bp, BGLAP Hs01587814_g1, Thermo Scientific) and the reference gene (eukaryotic elongation factor, EEF-1, Hs00265885_g1, Thermo Scientific) as well were quantified using the TaqMan Mater Mix and TaqMan probes. The fluorescence intensity was measured with the Light Cycler 480 from Roche (Basel, Switzerland). The thermo cycling parameters were 95 °C for 10 min; 95 °C for 10 s, 60 °C for 10 s (45 cycles); and 40 °C for 1 min. All samples were scaled relative to the median of the EEF1 expression level. The gene expression data were analyzed using the 2^(−ΔCt)^ method (relative quantification). Also, hMSCs prior to seeding on the scaffolds (day 0) and hMSCs cultured in differentiation medium seeded on the bottom of the wells (PS) were evaluated.

#### 2.6.6. Immunohistochemical Staining

Based on the previous results, PHB/PLA-SN and PHB/PLA-SN_TCP samples were selected for immunohistochemical staining. Osteocalcin and type I collagen were visualized using indirect immunofluorescence staining on days 14, 21, and 28 and on days 14 and 21, respectively. The samples were firstly rinsed in PBS and fixed with frozen methyl alcohol (−20 °C) for 10 min. Secondly, they were rinsed in PBS and incubated in the solution containing 0.1% of Triton-X-100 (Sigma Aldrich, St. Louis, MO, USA) and 1% of bovine serum albumin (BSA) in PBS. Afterwards, the samples were incubated over night at 4 °C with primary rabbit antibody in the case of osteocalcin (rabbit anti-osteocalcin IgG, Peninsula Laboratories, dilution 1:20, San Carlos, CA, USA) or mouse antibody in the case of type I collagen (mouse anti-collage type I IgG, DSHB, dilution 1:20, Iowa city, IA, USA) determination. Incubated samples were rinsed with PBS with 0.05% of Tween-20 (Sigma Aldrich, St. Louis, MO, USA) for 5, 10 and 15 min. Subsequently, the samples were rinsed in PBS and supplemented with respective secondary antibodies containing Alexa Fluor 488 fluorophore (goat anti-mouse, Biotech; goat anti-rabbit, LifeTechnologies, OR, USA). The incubation in the dark at laboratory temperature took 45 min. Rinsing with PBS with 0.05% of Tween-20 for 5, 10 and 15 min followed. Afterwards, the samples were rinsed with PBS, incubated for 5 min with propidium iodide (5 μg∙mL^−1^) and rinsed with PBS three times. The Zeiss LSM 5 DUO was used for imagining with the wavelengths set to *λ*_ex_ = 488 nm and *λ*_em_ = 520 nm for Alexa fluor 488 and *λ*_ex_ = 560 nm and *λ*_em_ = 580 nm for propidium iodide.

#### 2.6.7. Statistical Analysis

Data obtained from DNA test and MTS quantification were tested in the SigmaStat 3.5 software (Systat Software, Inc., San Jose, CA, USA). The one-way analysis of variance (ANOVA) and the Tukey/Student-Newman-Keuls methods were used. The level of significance was set at lower than 0.05 or lower than 0.001 (indicated by * in the graphs).

## 3. Results

### 3.1. Thermal Properties of Prepared Materials

**Figure 1:**
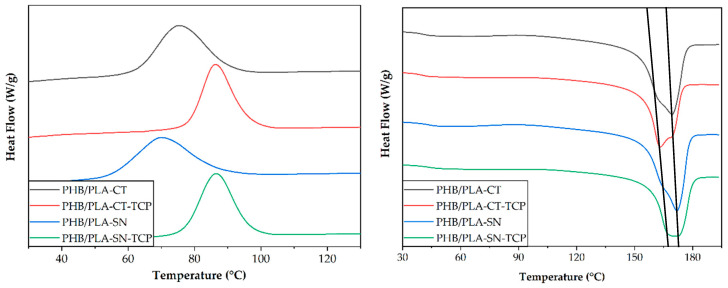
First cooling and second heating DSC scans of prepared materials.

The study of thermal properties of prepared materials was performed by DSC measurements. DSC scans of first cooling cycle and second heating cycle are depicted in Figure 1 and measured values are listed in Table 3.

As can be seen from the cooling scan, the crystallization temperature (PHB *T*_c1_) of both composite samples (PHB/PLA-CT_TCP and PHB/PLA-SN_TCP) is higher than in the case of non-filled plasticized PHB/PLA blend. The increase in crystallization temperature due to nucleation effect of TCP is from 76 to 86 °C for Citroflex and from 70 to 87 °C for Syncroflex. The melting curves of all samples exhibited a double peak typical for PHB. The Citroflex-based samples had lower melting temperature (PHB *T*_m2_) than the Syncroflex-based samples and the temperature decreased further with TCP addition to 163 °C for PHB/PLA-CT_TCP. The crystallinity calculated according to the equation (1) lying in the range of 56–61%, is the lowest for the sample PHB/PLA-CT_TCP.

In the blend of highly crystalline PHB and amorphous PLA, the plasticizer is expected to be located in the amorphous, PLA enriched phase. Therefore, the plasticizer is supposed to affect the glass transition of PLA (PLA *T*_g_). In all samples, the PLA *T_g_* was in the range of 41–43 °C, not affected by the presence of TCP.

### 3.2. Thermal Stability of Prepared Composites

**Figure 2:**
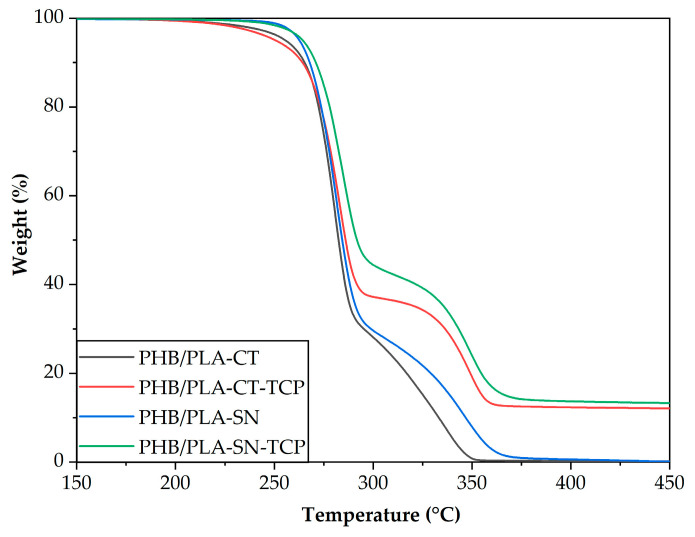
TGA curves of prepared samples.

The resulting TGA curves are shown in Figure 2 and the degradation temperatures in Table 4. As you can see from the figure, all samples had two-step decomposition, the first step corresponding to PHB and the second one to PLA decomposition. No visible step corresponding to the plasticizer is visible as both plasticizers decompose together with the polymers. However, at approximately 250 °C the samples with Citroflex show a greater weight loss than the samples with Syncroflex indicating that this plasticizer is more volatile.

The degradation temperature of neat PHB (PHB *T*_d_) is 286 °C, while in studied blends it varies from 281 to 284 °C. The degradation temperature of neat PLA (PLA *T*_d_) is 343 °C. In the case of PHB/PLA-CT sample, the temperature was lowered to 337 °C. The rest of the samples showed increased degradation temperature, up to 348 °C for both filled samples. It can be concluded that TCP does not act as a pro-degradant in PHB/PLA/plasticizer blends.

The amount of TCP determined by TGA was 11,9 wt.% for PHB/PLA-CT_TCP and 12.9 wt.% for PHB/PLA-SN_TCP. In the former, the deviation is attributed to the material inhomogeneity.

### 3.3. Optimal Printing Conditions and Printability

We managed to FDM 3D print the testing specimens from all prepared biocomposite filaments. Firstly, the TTT was executed. The geometric elements (colonnade, bridges, circle hole, overhangs) located on each floor of temperature tower printed with the nozzle temperatures ranging from 180 to 220 °C were visually evaluated (as described in [33]). This test provided us with the information about optimal nozzle temperature which was used for further printing tests. The optimization of flow parameter was executed as well, providing us with indirect information about the thickness of prepared filaments. The final optimized conditions can be seen in Table 5 together with the results of warping test. As can be seen from the table, the optimal temperature lies between 190–195 °C for all samples except for PHB/PLA-CT in which it is 5 °C lower. The optimal flow setting is 100% for all samples except for PHB/PLA-CT_TCP, which exhibited 90% probably due to the filament diameter slightly higher than 1.75 mm.

The warping coefficient was measured according to [33] for both temperatures identified by TTT. By definition, lower warping coefficient means lower warping during the printing. To give a basic idea about the meaning of specific values of warping coefficient, the commercial Beige PLA filament (Prusa Research, Prague, Czech Republic) has the warping coefficient of 2.9 ± 0.1 and therefore the goal is to reach comparable or lower values. Based on the results presented in Table 5, measured samples exhibited even lower warping coefficient in the range from 1.9 to 2.6 except for the sample PHB/PLA-B6_TCP. Both plasticized samples, PHB/PLA-CT and PHB/PLA-SN have comparable warping coefficient (2.0–2.2) favoring higher of the two tested temperatures. The addition of TCP led to the increase of unwanted warping phenomenon in the case of both plasticizers especially for higher printing temperature. However, this effect is much stronger for Citroflex samples as the PHB/PLA-CT_TCP as the only sample shows higher warping than commercial material. The warping coefficient of this sample was one third higher than for unfilled reference for the printing temperature of 190 °C. On the other hand, the warping level of PHB/PLA-SN_TCP sample for the printing temperature of 190 °C was comparable to the plasticizer unfilled samples. When using higher temperature, the printability worsened.

### 3.4. Tensile Properties of Prepared Biocomposites

The results of the tensile test of 3D printed standardized tensile testing specimens, 5A dogbones, can be seen in Table 6. The Young modulus (E modulus, E_T_) of PHB/PLA blend plasticized with Citroflex and Syncroflex is comparable within the measurement error of around 2.3–2.4 GPa. However, the tensile strength (*σ*_max_) of unfilled sample with Citroflex is lower than that of Syncroflex unfilled sample (32.2 ± 1.3 MP vs. 37.6 ± 3.4 MPa, respectively). Simultaneously, the elongation at break (*ε*_max_) for the sample PHB/PLA-CT is higher than in the case of PHB/PLA-SN but with greater deviation at the same time (22.6 ± 13.1% for Citroflex and 16.9 ± 3.0% for Syncroflex). When TCP as a bioactive filler is added to these blends, the modulus is slightly increased (the highest 2.6 ± 0.1 GPa value is achieved for PHB/PLA-CT_TCP) due to the addition of high-modulus fraction to the matrix. At the same time, the tensile strength drops, the decrease of approximately 12% and 15% from the original value for Citroflex and Syncroflex samples, respectively, is observed. This is attributed to the samples’ inhomogeneity caused by the presence of TCP crystals. 

As well, the elongation at break significantly decreased with the TCP addition in comparison to the unfilled samples. PHB/PLA-CT_TCP reaches only 15% of the value of elongation at break of unfilled PHB/PLA-CT sample. As for Syncroflex, this decrease is to 38% of original value. However, the sample PHB/PLA-SN_TCP reached the elongation at break of 6.4 ± 0.8%, which is almost twice the value of corresponding sample with citrate-based plasticizer causing probably unwanted reactions with TCP, which is acid-sensitive.

### 3.5. Flexural Properties of Prepared Materials

**Figure 3:**
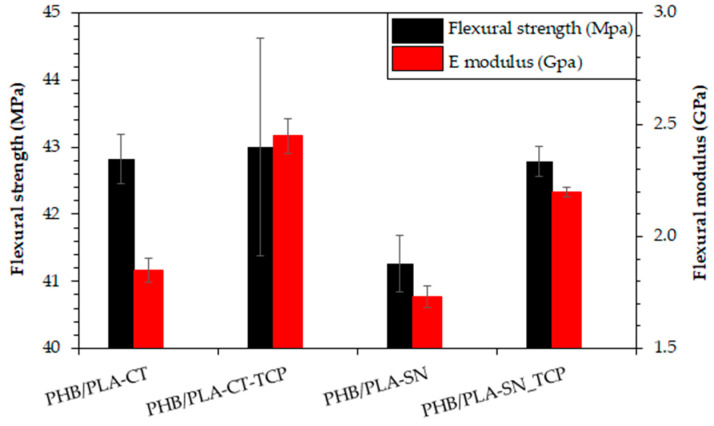
The results of flexural test of 3D printed specimen.

To observe the materials deformation behavior during bending, the flexural test was conducted on 3D printed rectangular testing specimen with results presented in Figure 3.

The flexural modulus is overall lower than the tensile modulus, being between 1.7–1.9 GPa for the samples without TCP. The reason for that is probably the fact that this modulus reflects the properties of only small specific locations on the surface of the sample. Significant increase is observed with the addition of TCP, up to 2.5 ± 0.1 GPa and 2.2 ± 0.0 GPa for the samples plasticized with Citroflex and Syncroflex, respectively. The flexural strength is comparable among all samples, ranging from 41.3 (PHB/PLA-SN) to 43.0 MPa (PHB/PLA-CT_TCP). As for Syncroflex samples, slight increase is observed with the addition of filler.

### 3.6. In Vitro Testing on Scaffolds

#### 3.6.1. Cell Proliferation and Metabolic Activity

To evaluate the cell proliferation on prepared scaffolds, the DNA quantification was performed. This test determined the overall number of cells proliferated on samples. The metabolic activity of cells was measured via the MTS assay.

**Figure 4:**
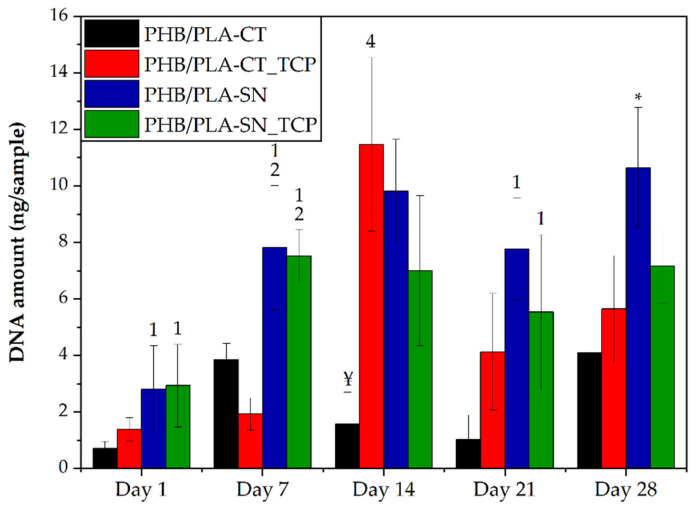
Cell proliferation. The number above the column indicates statistically significantly higher values compared to the column of that number, *p* < 0.05. * means the statistically highest value of all groups for a given day, ¥ means the statistically lowest value of all groups for a given day.

The results of DNA quantification are depicted in Figure 4. On day 1, the cell adhesion was statistically higher in the samples containing Syncroflex compared to the samples with Citroflex plasticizer. The sample PHB/PLA-CT had very low amount of DNA until day 28 of the experiment and seems to be worse in terms of its biocompatibility. The sample containing Citroflex plasticizer and TCP, PHB/PLA-CT_TCP, had low DNA values on days 1 and 7, followed by an increase on day 14. In the following days however, the number of cells decreased again. On the samples containing Syncroflex, cell proliferation occurred mainly during the first seven days, after which the values were rather stable. The highest amount of DNA was observed in the late phase of the experiment, especially for the sample PHB/PLA-SN.

**Figure 5:**
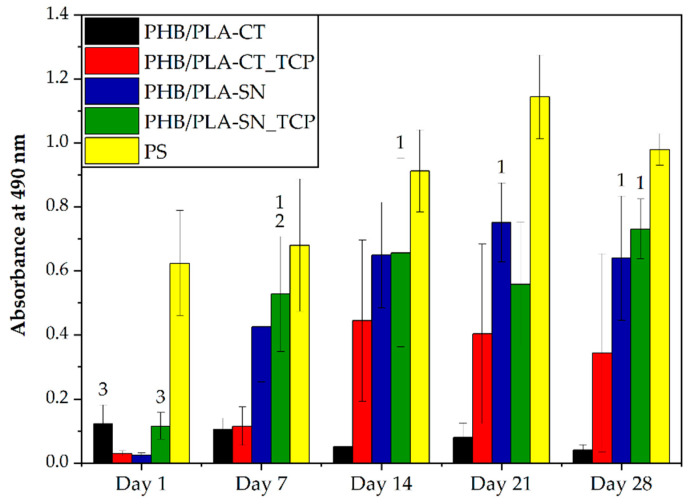
Metabolic activity of cells on the scaffolds. The numbers above the column indicate statistically significantly higher values compared to the column of that number, *p* < 0.05.

The results of metabolic activity measurement are shown in Figure 5. On the first day of the experiment, the samples PHB/PLA-CT and PHB/PLA-SN_TCP showed the highest metabolic activity but still much lower than the control PS plastic showed. For the rest of the experiment however, the sample PHB/PLA-CT had the lowest metabolic activity of all samples, which might be due to the citrate-based plasticizer and some of its releasing residues. The values were generally lower for the samples containing Citroflex plasticizer, than for those with Syncroflex. However, the TCP addition to citrate-based samples improving the cell metabolic activity especially in the later stages might be probably due to the neutralizing effect of the basic released TCP interacting with possible acid-residues from Citroflex. Regarding the samples with Syncroflex, the values of metabolic activity were higher or comparable for the sample containing TCP than for the sample without bioactive filler proving no unwanted side reactions between the plasticizer and TCP. Moreover, from day 7 the values of absorbance are not statistically different from a control plastic.

To sum up, the result of the MTS assay was similar to the result of DNA quantification showing higher cell proliferation and viability on the samples with Syncroflex compared to those with CT.

**Figure 6:**
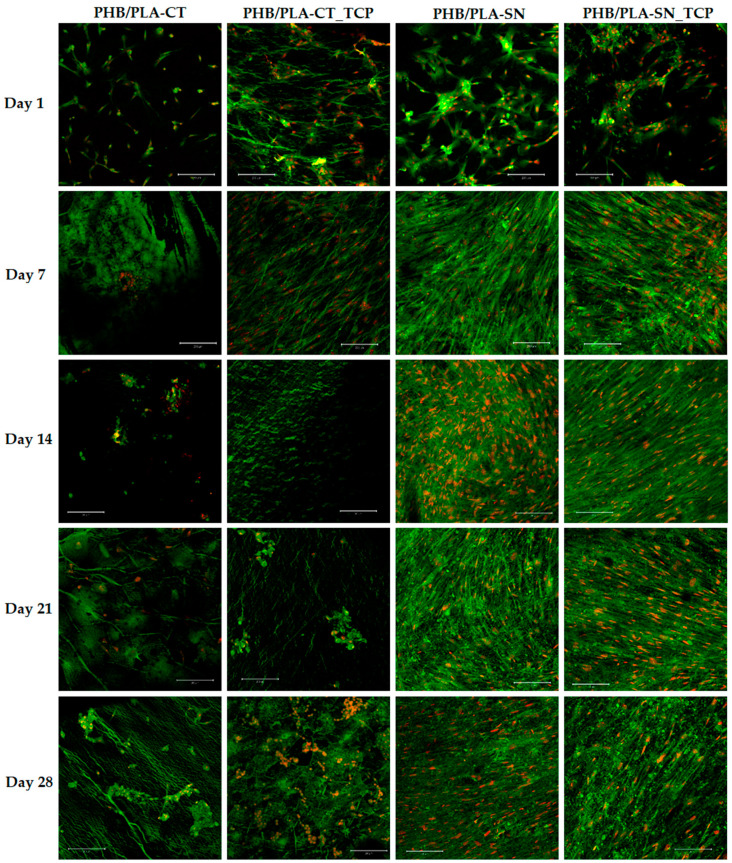
hMSCs on scaffolds from tested materials, magnification 100×, the bar is 200 µm.

To gain comprehensive information, also the number and the shape of the cells on scaffolds were observed using confocal microscopy. The results can be seen in Figure 6. The difference between Citroflex and Syncroflex samples is prominent, confirming the results of MTS test and DNA quantification.

On day 1 of the test, the highest number of cells forming a thick network over most of the area of the scaffold was observed on the sample PHB/PLA-CT. Nevertheless, from day 7 until the end of the experiment only a few cells were observed in the case of this sample. The cells formed clusters and they all had round shape indicating their poor condition not suitable for their proliferation. The sample with Citroflex plasticizer and TCP, PHB/PLA-CT_TCP, showed high number of cells during the first and seventh days of the experiment, but after that the situation was similar to the case of its filler-free reference.

In contrast, a profound increase of the number of cells was visible on the seventh day of the experiment for both samples with Syncroflex. From then on, the cells formed a confluent layer. For some samples, this layer peeled off due to the smooth surface of the scaffold.

#### 3.6.2. Osteogenic Differentiation of Cells

The osteogenic differentiation of hMSCs on scaffolds was monitored by ALP activity and real-time PCR analysis in all samples, and by immunohistochemical staining only in the samples containing Syncroflex as the samples containing Citroflex had insufficient number of cells on the scaffolds.

**Figure 7:**
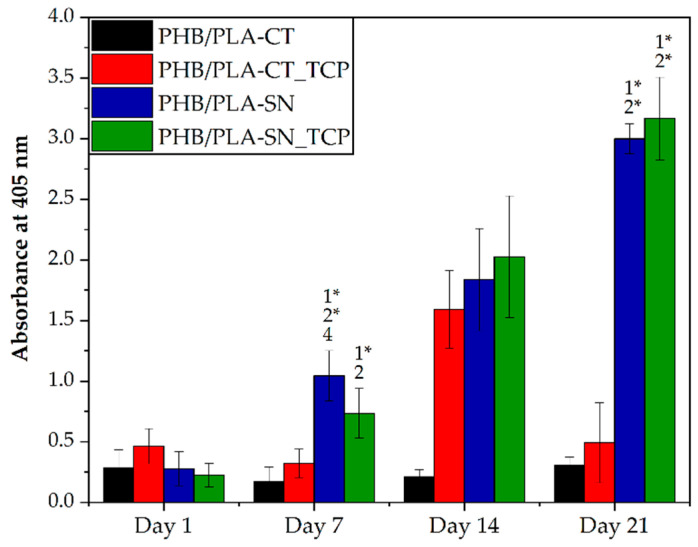
The results of ALP assay. The numbers above the column indicate statistically significantly higher values compared to the column of that number for *p* < 0.05 and *p* < 0.001 with *.

Figure 7 depicts the results of ALP assay of tested materials. On day 1, the values were low and comparable among all samples. The following days, the sample PLA/PHB-CT containing citrate-based plasticizer remained on its original value. After the TCP addition the amount of ALP reached the maximum on day 14 and then dropped again to the initial value on day 21. The samples with Syncroflex exhibited statistically higher values of produced ALP than the samples with CT, especially on day 21 of the experiment, where the difference is profound.

**Figure 8:**
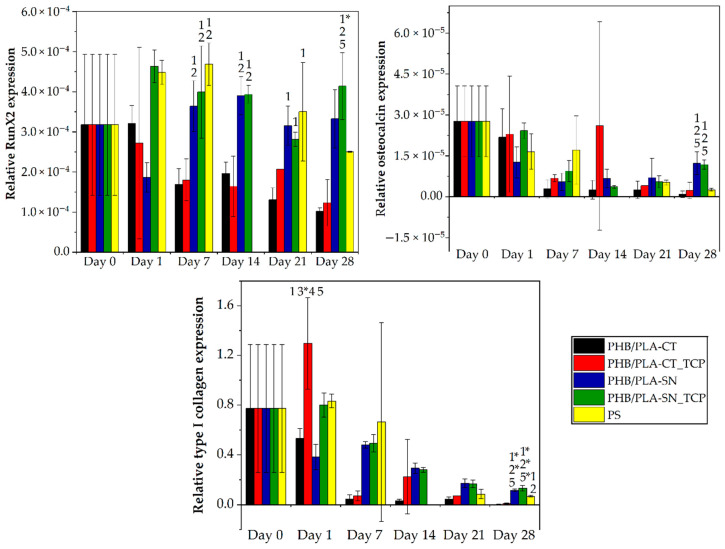
Gene expression of osteogenic markers RunX2, collagen I and osteocalcin. The numbers above the column indicate statistically significantly higher values compared to the column of that number for *p* < 0.05 and *p* < 0.001 with *.

The expression of typical osteogenic markers was monitored via real-time PCR analysis and is shown in Figure 8.

RunX2, the marker of early osteogenic differentiation, was expressed in comparable quantity during the experiment for individual samples. The highest expression level was observed for the samples with Syncroflex, PHB/PLA-SN and PHB/PLA-SN_TCP. The expression of both type I collagen and osteocalcin, the marker of late osteogenic differentiation, dropped during the experiment for all samples. In the late stage of the analysis (days 21 and 28), the highest amount of expressed type I collagen was detected again in the samples containing Syncroflex. Regarding osteocalcin, no statistically significant difference was observed until the 28th day of the test, when Syncroflex samples showed the highest values.

**Figure 9:**
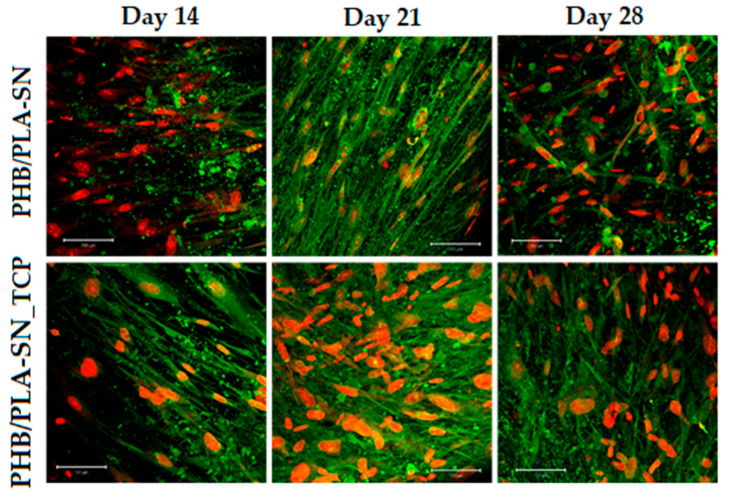
Osteocalcin staining, cell nuclei are red and osteocalcin green, magnification 200×, the bar is 100 µm.

**Figure 10:**
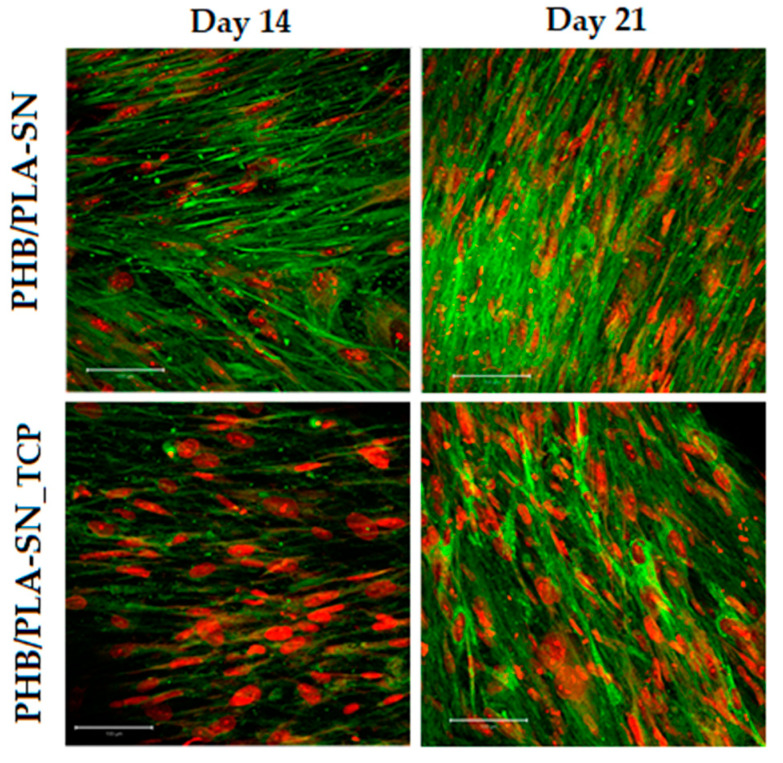
Collagen I staining, cell nuclei are red and collagen is green, magnification 200×, the bar is 100 µm.

Although rather low levels of expression of osteocalcin and type I collagen were measured by RT-PCR analysis, both markers could be visualized by confocal microscopy after immunohistochemical staining (see Figure 9 for osteocalcin and Figure 10 for type I collagen). Both proteins were detected for both studied samples, PHB/PLA-SN and PHB/PLA-SN_TCP, on all monitored days.

## 4. Discussion

Two series of samples based on poly(3-hydroxybutyrate) (PHB) and poly(d,l-lactide) (PLA) blend were compounded, one with *n*-Butyryl tri-*n*-hexyl citrate (Citroflex, CT) and one with oligomeric adipate ester (Syncroflex, SN) as a plasticizer. For each plasticizer, unfilled reference sample and composite sample with 13 wt.% of tricalcium phosphate (TCP) were prepared. Therefore, both the difference between the performance of these two plasticizers and the effect of bioactive filler addition were studied.

The difference of used plasticizers from the viewpoint of thermal properties of resulting materials observed by Differential Scanning Calorimetry (DSC) is negligible. The Citroflex samples exhibit lower melting temperature of PHB, the glass transition temperature of PLA being around 42 °C is comparable for all samples. TCP acts as a nucleating agent increasing the temperature of crystallization of about 10–15 °C. Moreover, the addition of TCP does not decrease the thermal stability of the blend, contrariwise the temperature of maximum degradation rate of both polymers is higher for both composite samples than for their unfilled reference as measured by Thermogravimetric analysis.

All samples were successfully extruded to the form of 3D printing filaments with the precise diameter of 1.7–1.8 mm followed by Fused Deposition Modeling 3D printing of testing specimen for subsequent test. To ensure the highest quality of printed objects, two crucial 3D printing parameters, the nozzle temperature and the flow were optimized by the temperature tower test (TTT) and the filling study, respectively. The printability of these blends was also evaluated applying new, recently published Warping Test (see [33]). The optimum printing temperature range obtained from TTT was in the range of 190–195 °C for all samples except for PHB/PLA-CT, for which it was slightly lower, 185–190 °C, corresponding to the results from DSC measurement. Lower melting and processability temperatures of this sample indicate more profound plasticizing effect of Citroflex than Syncroflex. 

The warping test further specified the optimum printing temperature, generally being the higher one of the two selected by TTT for unfilled samples and the lower temperature of the two for the samples filled with TCP. The composite sample with Citroflex plasticizer showed the highest level of warping of all tested samples. On top of that, this sample showed even higher warping levels than commercial PLA Prusa filament. On the contrary, the composite sample with Syncroflex reached the same printability as unfilled reference at the printing temperature of 190 °C. All Syncroflex samples achieved better warping properties than commercial Prusa PLA filament subjected to identical testing conditions.

The mechanical testing confirmed Citroflex to have better plasticizing effect on studied PHB/PLA blend. The samples with Citroflex had lower tensile Young modulus, tensile strength and at the same time higher elongation at break than corresponding samples with Syncroflex. The TCP addition influenced the tensile modulus very slightly (E modulus increased from 2.3–2.4 to 2.5–2.6 GPa). At the same time, the tensile strength was reduced by about 12–15% to values close to 30 MPa. In the case of our samples, the tensile strength also corresponds with the yield strength and therefore its change cannot be explained by higher probability of cracking with the addition of filler. We attribute this effect to an imperfect adhesion between the polymer matrix and the filler, which manifests itself more profoundly in the tensile test, where the bulk properties are measured. In addition, the elongation at break drops with the addition of TCP to 3.4% for Citroflex and to 6.4% for Syncroflex sample. The flexural modulus of unfilled samples is lower than the tensile modulus, reaching the values of 1.7–1.9 GPa and is profoundly increased with the addition of the filler. Opposite to this, the flexural strength is comparable for all tested samples. To put these values into context, although the mechanical properties of bones are highly age and body part dependent, the modulus of compact bone in longitudinal direction (corresponds to measured values from tensile test) is reported to be 17.9 ± 3.9 GPa and the strength of 135 ± 15.6 MPa [6]. This 7-fold difference in the tensile modulus and 4.5-fold in the tensile strength represents a huge space for future development. The increase in these parameters can be achieved for example by varying the composition of the blends—lower amount of plasticizer with simultaneous higher amount of the filler; or by tuning the structure of 3D printed object.

Moreover, the set of in vitro tests of proliferation and viability and also the osteogenic differentiation of human mesenchymal stem cells (hMSC) on scaffolds prepared from studied materials was executed. The scaffolds were disks of 6 or 10 mm diameter and 1 mm thickness shape cut from compression molded plates.

Overall, the samples with Syncroflex showed superior performance compared to the samples with CT. The sample PHB/PLA-CT had consistently the worst results of all performed in vitro tests albeit showing promising properties for 3D printing and good mechanical properties. The cell growth on this sample was almost non-existent and observed cells had round shape and existed in clusters indicating their bad condition. The alkaline phosphatase (ALP) activity was minimal during all days of the test. RunX2, a marker of osteogenic differentiation, was detected, but in the lowest amount of all samples and osteocalcin and collagen were measured only the first day of the experiment. Mildly better results were observed in the case of PHB/PLA-CT_TCP, its corresponding filled sample. The number of metabolically active cells was higher than that for PHB/PLA-CT, but also with the higher measurement error. As observed by microscope, although the number of cells was higher, their shape and overall condition were poor. The ALP activity showed an increase up to the 14th day of the experiment and then a drop followed. On day 21 of the experiment, the values of both Citroflex samples were very close to each other. The expression of osteogenic markers for PHB/PLA-CT_TCP was extremely low and again comparable to the sample PHB/PLA-CT. To conclude, the Citroflex plasticizer has the potential to be used for producing blends suitable for 3D printing. Nevertheless, these tests proved low biocompatibility of such material hence being inappropriate for the medical applications in human body. 

The Syncroflex-based samples on the other hand demonstrated excellent cell proliferation during the entire course of the test, with PHB/PLA-SN with the most cellular DNA detected. The same trend was observed for metabolically active cells. The visualization using confocal microscope revealed that the cells formed a confluent layer and had a natural elongated shape proving their good condition. No major difference between the two Syncroflex samples was detected by these tests. The same applies when it comes to the measurement of ALP activity, both samples, PHB/PLA-SN and PHB/PLA-SN_TCP, exhibited a continuous increase throughout the test reaching comparable values on the last day. The expression of RunX2 for the sample PHB/PLA-SN_TCP reached higher value than the control on the 28th day of the test. The levels of expression of osteocalcin and type I collagen were rather low, but these proteins were visualized by immunohistochemical staining and were present during all tested days. Syncroflex proved to be a suitable plasticizer for PHB/PLA blend. Both samples, unfilled reference and prepared composite were not only suitable for the 3D printing applications but also demonstrated excellent results of in vitro tests which shows their potential for use as the material for stem cell-seeded scaffolds for regenerative medicine of bones.

## 5. Conclusions

To conclude, *n*-Butyryl tri-*n*-hexyl citrate (CT) plasticizer is a suitable plasticizer for poly(3-hydroxybutyrate) (PHB)/poly(d,l-lactide) (PLA) blends leading to improved processability and mechanical properties. However, the samples with Citroflex showed poor biocompatibility properties in in vitro test excluding them from the future research in the field of tissue engineering.

Both studied materials containing PHB/PLA and Syncroflex plasticizer, with and without the tricalcium phosphate (TCP) addition, are suitable for the 3D printing by FDM method and at the same time exhibit promising properties to be used as materials for bone tissue scaffolds. However, the in vitro testing revealed that the scaffold design needs to be adjusted mainly by means of surface topography. Therefore, further research will take place in the direction of tuning the surface and mechanical properties of developed blends.

## Figures and Tables

**Table 1:** The composition of prepared samples: the basic mixture is composed of 12% of plasticizer and the remaining 88% is PHB/PLA in the ratio of 70/30. For filled samples, 15 wt.% of TCP is added to the mixture.

	Amount (wt.%)				
Sample	PHB	PLA	Citroflex	Syncroflex	TCP
PHB/PLA-CT	62	26	12	0	0
PHB/PLA-CT_TCP	54	23	10	0	13
PHB/PLA-SN	62	26	0	12	0
PHB/PLA-SN_TCP	54	23	0	10	13

**Table 2:** Basic printing parameters of used 3D printer.

Filament diameter	1.75 mm
Nozzle diameter	0.4 mm
Layer height	0.2 mm
Width of printed layer	0.45 mm
Perimeter printing speed	45 mm∙s^−1^
Fill print speed	200 mm∙s^−1^
Bed temperature	20 °C
Cooling fan power	100%

**Table 3:** Thermal properties of tested materials.

	Glass Transition Temperature PLA *T*_g_ (°C)	Crystallization Temperature PHB *T*_c1_ (°C)	Melting Temperature PHB *T*_m2_ (°C)	Crystallinity PHB *X*_c1_
PHB/PLA-CT	42.8	75.6	169.3	59.9
PHB/PLA-CT_TCP	41.1	86.3	163.3	56.7
PHB/PLA-SN	40.8	69.8	171.9	61.1
PHB/PLA-SN_TCP	42.3	86.6	169.9	59.1

**Table 4:** The results of TGA.

	Maximum Rate of Degradation		Residue Amount *w*% at 600 °C
	PHB *T*_d_ (°C)	PLA *T*_d_ (°C)	
PHB/PLA-CT	281	337	-
PHB/PLA-CT_TCP	283	348	11.9
PHB/PLA-SN	281	346	-
PHB/PLA-SN_TCP	284	348	12.9

**Table 5:** Optimized printing conditions and the results of warping test.

	Optimal Nozzle Temperature (°C)	Optimal Flow (%)	Warping Coefficient (-)		
			185 °C	190 °C	195 °C
PHB/PLA-CT	185–190	100	2.2 ± 0.1	2.0 ± 0.1	
PHB/PLA-CT_TCP	190–195	90		3.0 ± 0.2	3.2 ± 0.3
PHB/PLA-SN	190–195	100		2.1 ± 0.1	2.0 ± 0.1
PHB/PLA-SN_TCP	190–195	100		2.2 ± 0.2	2.5 ± 0.1

**Table 6:** The results of tensile test.

	Young’s Modulus E_T_ (GPa)	Tensile Strength *σ*_max_ (MPa)	Elongation at Break *ε*_max_ (%)
PHB/PLA-CT	2.3 ± 0.1	32.2 ± 1.3	22.6 ± 13.1
PHB/PLA-CT_TCP	2.6 ± 0.1	28.3 ± 0.8	3.4 ± 2.0
PHB/PLA-SN	2.4 ± 0.2	37.6 ± 3.4	16.9 ± 3.0
PHB/PLA-SN_TCP	2.5 ± 0.0	32.1 ± 0.7	6.4 ± 0.8
